# Prognostic Nomogram For Locoregionally Advanced Nasopharyngeal Carcinoma

**DOI:** 10.1038/s41598-020-57968-x

**Published:** 2020-01-21

**Authors:** Yanming Jiang, Song Qu, Xinbin Pan, Shiting Huang, Xiaodong Zhu

**Affiliations:** Department of Radiation Oncology, Affiliated Tumor Hospital of Guangxi Medical University, Cancer Institute of Guangxi Zhuang Autonomous Region, Nanning, Guangxi China

**Keywords:** Cancer, Head and neck cancer

## Abstract

The TNM staging system of NPC is the most important model for survival prediction. However, this model does not consider the biological variability of the tumor itself. This study aimed to develop a nomogram for predicting the overall survival of loco-regionally advanced nasopharyngeal carcinoma. 487 Patients with confimed nasopharyngeal carcinoma who underwent IMRT and chemotherapy were included in this study. We established prognostic nomogram for overall survival (OS) based on the Cox proportional hazards model. The predictive accuracy and discriminative ability were measured using the concordance index (C-index) and calibration curve. Nomogram was validated externally by assessing discrimination and calibration using an independent data set. Continuous net reclassification improvement (NRI) and integrated discrimination improvement (IDI) were used to analyze whether nomogram improve the prediction of survival than TNM stage system. Recursive partitioning analysis (RPA) was performed to stratifying risk of patients. Age, T-stage, N-stage, NLR, LDH were included in the nomogram for OS. The C-index of the nomogram for OS were 0.726 (95% CI, 0.690 to 0.762); The calibration curve showed the nomogram was able to predict 5-year OS accurately. The nomogram had a higher C-index than the TNM stage system (0.726 VS 0.632, P-value < 0.001). The NRI was 0.235 (95% CI: 0.129 to 0.396, P < 0.001), the IDI was 0.079 (95% CI: 0.034 to 0.396, p < 0.001). RPA was performed to stratify patients into three risk group, OS was significantly different between all three risk groups. High risk groups can be benefited survival from adjuvant chemotherapy. The nomogram outperformed the TNM staging system in predicting the OS of loco-regionally advanced nasopharyngeal carcinoma underwent intensity modulated radiation therapy and chemotherapy.

## Introduction

Nasopharyngeal carcinoma (NPC) is endemic to the south of China and Asia; an NPC incidence of 2 per million people in China has been reported^1^. More than 70% of the newly diagnosed cases are classified as locoregionally advanced disease^2^. Concurrent chemoradiotherapy is the standard treatment for locally advanced nasopharyngeal carcinoma^3^. However, in some patients, the disease progresses within a few years after chemoradiotherapy. Therefore, identifying a prognostic model for early progression would allow for a better therapeutic plan.

The TNM staging system of NPC is the most important model for survival prediction. However, this model does not consider the biological variability of the tumor itself. The prognosis of patients at the same stage receiving the same treatment varies greatly. Therefore, another prognostic model, based on the TNM staging system combined with other prognosis factors, has been evaluated^4^; however, this model was not validated and has limited clinical applicability. Therefore, in this study, we established and validated a nomogram for locoregionally advanced NPC patients who received intensity-modulated radiation therapy (IMRT) and chemotherapy.

## Results

### Baseline characteristics of the patients

A total of 487 cases were included in this research. According to the ratio of 3:1, all of the cases were randomly divided into the primary cohort (n = 365) and the validation cohort (n = 122). Twenty-six patients underwent radiotherapy without chemotherapy due to advanced age or other reasons; 146 patients underwent concurrent chemoradiotherapy (CCRT) and 315 patients underwent adjuvant chemotherapy (AC) following CCRT. The baseline characteristics between the two cohorts were not significantly different (Table 1).

**Table 1:** Baseline characteristics of the patients.

characteristic	Primary cohort (No. of patients)	Validation cohort (No. of patients)	X2	P value
No.	365	122		
Age			0.433	0.511
<50 years	201	63		
≥50 years	164	59		
Sex			0.914	0.339
male	279	88		
female	86	34		
Stage			0.361	0.548
III	183	65		
IV	182	57		
T-stage			2.748	0.097
T1-2	96	23		
T3-4	269	99		
N-stage			0.000	0.989
N0-1	84	28		
N2-3	281	94		
Adjuvant chemotherapy			3.354	0.067
yes	230	88		
no	135	34		

### Survival outcomes

The median follow-up time was 55.7 months (range 3.09–91.7 months). The 1-year, 3-year, and 5-year overall survival (OS) rate were 98%, 87%, and 82%, respectively. The median follow-up time in the primary cohort was 55.5 months (range 3.42–91.6 months), and the 1-year, 3-year, and 5-year OS rate were 98%, 87% and 82%, respectively. At the end of follow-up, there were 64 deaths, of those, 50 cases have the tumor-related deaths. The median follow-up time in the validation cohort was 56.4 months (range 3.09–91.7 months), and the 1-year, 3-year, and 5-year OS rate were 98%, 88% and 82%, respectively. At the end of follow-up, 22 patients died, of those, 15 patients died of tumor-related causes.

### Independent prognostic factors in the primary cohort

Univariate analysis showed that age, stage, T-stage, N-stage, peripheral neutrophil–lymphocyte ratio (NLR), lactic dehydrogenase (LDH), and serum albumin (ALB), were associated with OS. Multivariable analyses continued to demonstrate that age, T-stage, N-stage, NLR, and LDH were independent prognostic factors for OS (Table 2). Proportional hazards assumptions were tested and found to be appropriate.

**Table 2:** Univariate and multivariate analysis of the primary cohort.

variable	OS (univariate analysis)		OS (multivariate analysis)	
	X2	P value	HR (95% CI)	P value
Sex	1.354	0.245	N/A	N/A
Age	14.573	<0.001	2.384 (1.399–4.061)	0.001
Stage	7.053	0.008	1.422 (0.820–2.464)	0.210
T12/T34	7.296	0.007	2.397 (1.089–5.273)	0.030
N01/N23	4.328	0.036	2.833 (1.329–6.036)	0.007
NLR	12.172	<0.001	1.908 (1.145–3.177)	0.013
LDH	8.438	0.004	2.437 (1.271–4.634)	0.007
ALB	8.332	0.004	0.614 (0.364–1.035)	0.067
				N/A
SF	0.331	0.565	N/A	
Adjuvant chemotherapy	1.582	0.209	N/A	N/A

N/A: Not applicable.

### Development of a nomogram for OS

**Figure 1:**
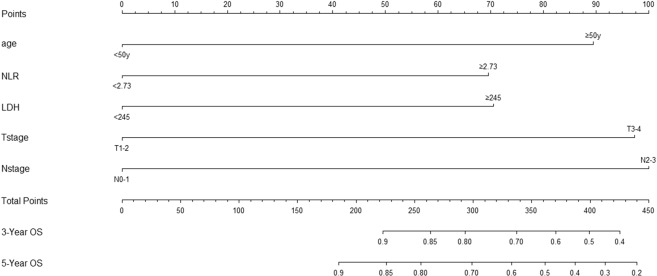
Prognostic nomogram of survival probabilities at 3-year and 5-year in patients with NPC.

**Figure 2:**
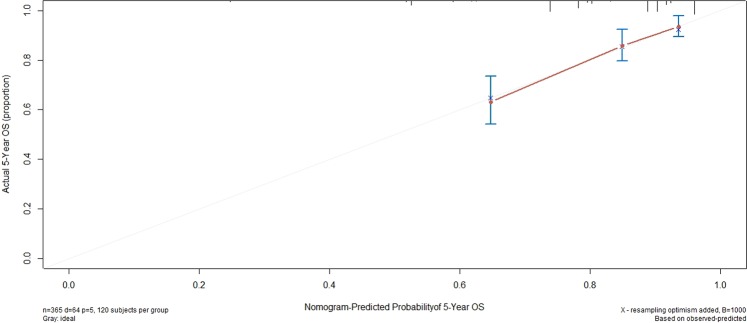
Calibration plots of survival probabilities at 5-year in patients with NPC.

On the basis of the multivariate analysis results, the OS prognostic nomogram was built using R 3.4.2 with the survival and rms package (Fig. 1). Within these variables, each subtype assigns a score on the score table (Table 3). By adding up the total score and positioning it on the total scale, we can easily draw a line to determine the probability of survival at each point in time. The C-index of the nomogram for OS was 0.726 (95% CI, 0.690 to 0.762). The akaike information criterion (AIC)value of the nomogram for OS was 695.15. Calibration plots revealed superb agreement between the nomogram predicted probabilities and the actual observations of 5-year OS (Fig. 2).

**Table 3:** Point assignment from nomograms and prognostic score.

Characteristics	Primary cohort (No of patients)	validation cohort (No of patients)	score	5-year OS
**NLR**				
≥2.73	109	35	69	75%
<2.73	256	87	0	85%
**LDH**				
≥245	34	12	70	67%
<245	331	110	0	84%
**Age**				
≥50years	164	59	89	74%
<50years	201	63	0	89%
**Tstage**				
T1-2	96	23	0	88%
T3-4	269	99	97	80%
**Nstage**				
N0-1	84	28	0	85%
N2-3	281	94	100	81%
**OSstatus**				
live	301	100		
dead	64	22		
**Risk groups**				
Low risk	81	19	<216	96%
Intermediate risk	250	84	216–306	81%
High risk	34	19	≥306	60%

### Validation of the nomogram for OS

**Figure 3:**
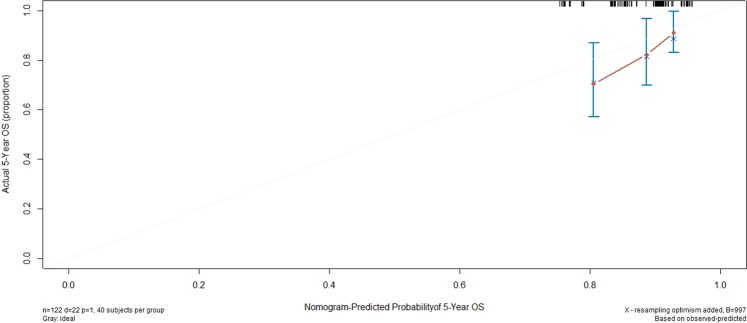
External validation of the nomogram to predict 5-year OS likelihoods in patients with NPC in the validation cohort.

The nomogram was externally validated in the validation cohort by computing the bootstrap C statistic and calibration plot. The C-index of the nomogram for predicting 5-year OS was 0.646 (95% CI, 0.534 to 0.759), The calibration curve indicated that the nomogram was well calibrated; the 5-year OS showed an optimal agreement between the actual observations and the nomogram prediction (Fig. 3).

### Comparison of predictive accuracy for OS between the nomogram and TNM staging system

**Figure 4:**
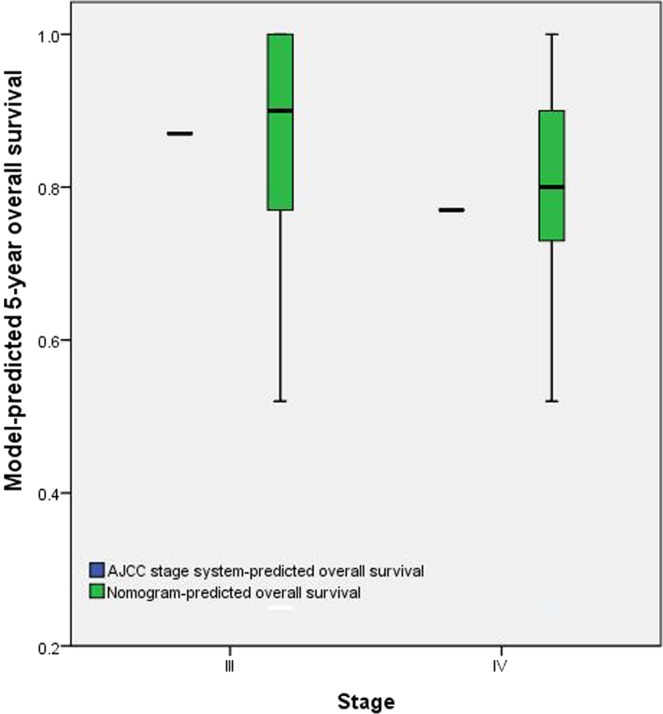
Distribution of nomogram-predicted 5-year overall survival within each AJCC stage grouping.

The TNM staging systems showed good prognostic stratification for patients. The C-index for the TNM staging system was 0.632 (95% CI: 0.599 to 0.665), and the AIC value of TNM staging system for OS was 717.31. The C-index for the TNM staging system was significantly lower than the C-index for the nomogram (0.726, P < 0.001). By the proposed nomogram, a wider range of predicted survival than AJCC staging system could be clearly identified within each TNM categories (Fig. 4). To determine whether or not the nomogram resulted in better prediction than the TNM staging system, we calculated both the NRI and IDI; the NRI was 0.235 (95% CI: 0.129 to 0.396, P < 0.001), and the IDI was 0.079 (95% CI: 0.034 to 0.396, p < 0.001).

### Performance of the nomogram in stratifying risk of patients

**Figure 5:**
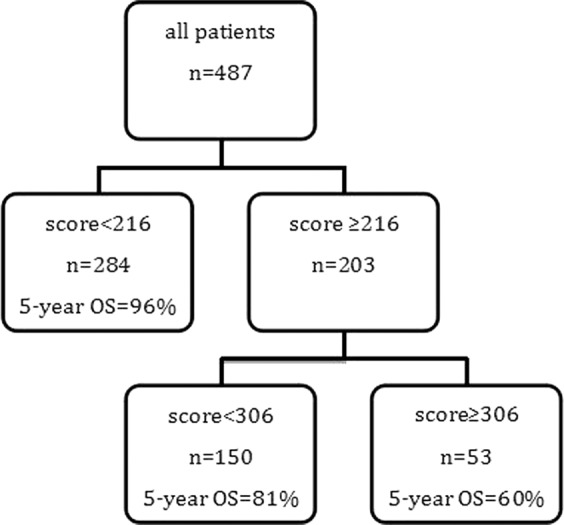
RPA-generated risk stratification of patients with NPC for predicting 5-year OS.

The nomogram for OS had good predictive value. On the basis of the scores estimated from the developed nomogram for OS, the whole series of patients could be categorized into 3 risk groups by a recursive partitioning analysis (RPA) (Fig. 5). The 5-year OS rate for the low, intermediate, and high-risk groups were 96%, 81%, 60%, respectively (P < 0.05). In the subgroup analysis, we found that, compared with concurrent radiochemotherapy, adjuvant chemotherapy had significantly improved survival in high-risk group patients (P = 0.022) (Fig. 5).

## Discussion

In this research, we developed a nomogram for locoregionally advanced NPC patients who underwent IMRT and chemotherapy. This nomogram aimed to estimate the probability of 5-year OS based on a multivariate Cox proportional hazards model that included five clinical variables. There are several prognostic nomograms for NPC^4–9^. Tang’s^5^ research developed a nomogram with or without EBV DNA for disease-free survival (DFS) prediction using the variables of age, sex, body mass index (BMI), T-stage, N-stage, pretreatment hypersensitive C-reactive protein (hs-CRP), LDH, hemoglobin levels and plasma epstein-barr virus (EBV) DNA, but only 34% of patients were treated with IMRT. CRP is a type of acute-phase protein and is a sensitive but nonspecific inflammatory marker. Recently, the combination of CRP and albumin has been used to develop the Glasgow prognosis scoring system (GPS) for the study of tumor prognosis. Yang^6^ also developed a nomogram for OS and DMFS based on the variables of age, sex, LDH, CRP, T-stage, N-stage, and EBV DNA. Similar to Tang’s research, radiotherapy treatment was mixed with conventional radiotherapy, three-dimensional conformal radiotherapy (3DCRT) and IMRT. Wu *et al*.^10^ found a significant correlation between elevated CRP levels and reduced OS, and developed a new N-C model for predicting survival based on CRP level and N stage. However, the model has not yet been validated. In addition, CRP is not routinely measured before treatment. Therefore, the clinical applicability of the prognostic models containing CRP is limited. Wu^7^ developed a nomogram based on the UICC 2002 TNM staging system for OS in NPC patients who received IMRT; however, this nomogram did not specifically provide a treatment decision, only a means to evaluate individual patient outcomes after IMRT.

In certain types of cancer, a nomogram has been developed and shown a more accurate prognosis than the traditional TNM staging system. The TNM staging system considers only anatomical information, and it is far from individualized risk stratification and determining precise therapeutic guidelines for targeted patients. We established a prognostic nomogram for OS, combining all confirmed prognostic factors (age, LDH and NLR), which had better prognostic efficiency than the TNM staging system and had a higher C-index and lower AIC value. To compare different prediction models, the improvement in discrimination can be assessed by quantifying an incremental value such as the change in the C-index. All of the published comparisons between nomograms for NPC and the TNM staging system were based on the C-index. Recently, a number of new measures for quantifying the added value from new markers were proposed, including the IDI and the NRI. These two measures have drawn much attention in the medical research, especially in the evaluation of markers for cardiovascular disease progression^11–13^.

We used the NRI and IDI to quantify the improved survival prognostication for all confirmed prognostic factors incorporated into the TNM staging system. The incorporation of age, LDH, and NLR into the TNM staging system resulted in an NRI of 0.444 (95% CI: 0.209 to 0.661, P = 0.01), and an IDI of 0.073 (95% CI: 0.022 to 0.129, P < 0.01). These statistics indicate that the nomogram improved the prognostic value compared to the TNM staging system. To the best of our knowledge, this study is the first attempt to quantify the improved survival prognostication of a nomogram compared to the TNM staging system.

**Figure 6:**
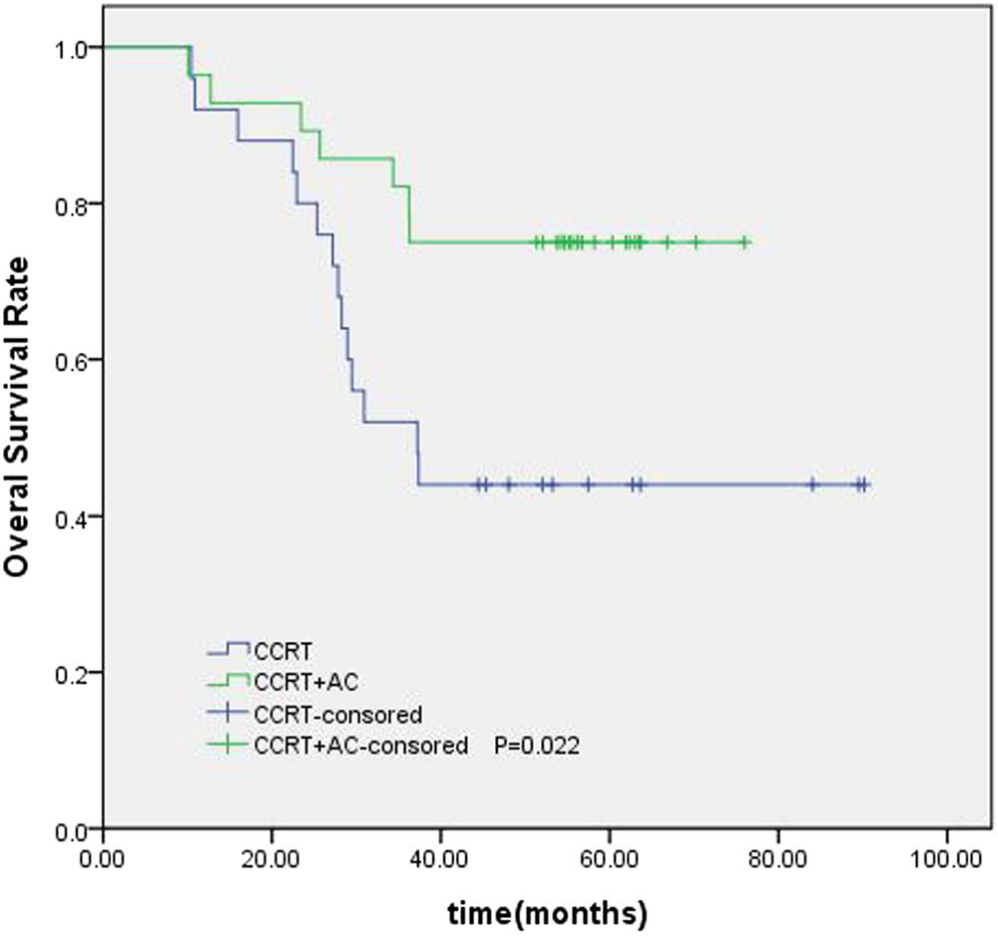
Kaplan-Meier OS curves for high-risk group patients with NPC.

**Figure 7:**
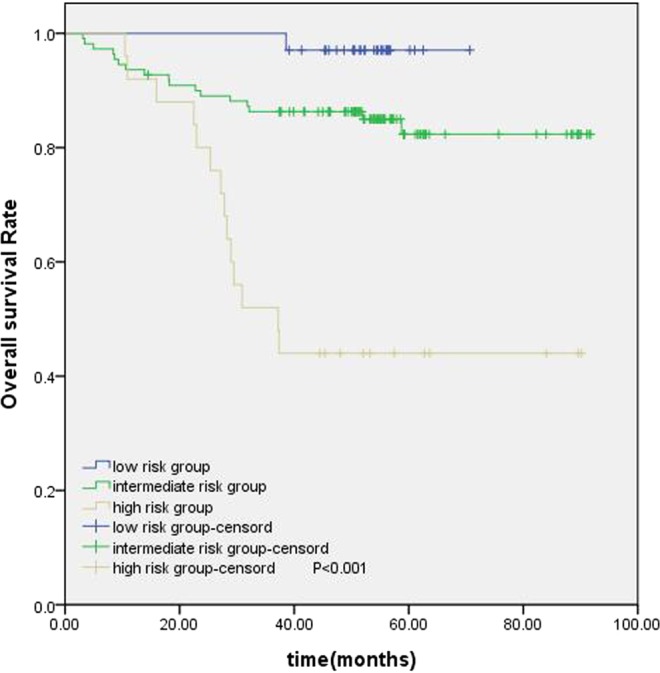
Kaplan-Meier survival curves for OS in the CCRT-along groups of low, intermediate and high risk.

**Figure 8:**
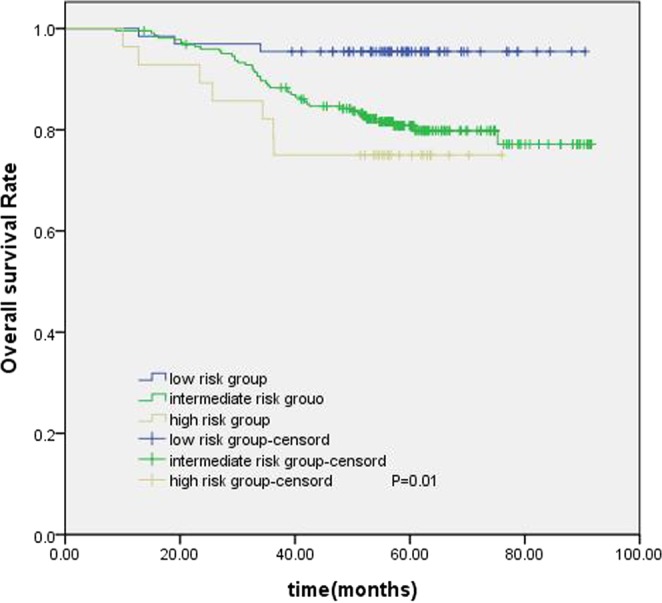
Kaplan-Meier survival curves for OS in the CCRT + AC groups of low, intermediate and high risk.

**Figure 9:**
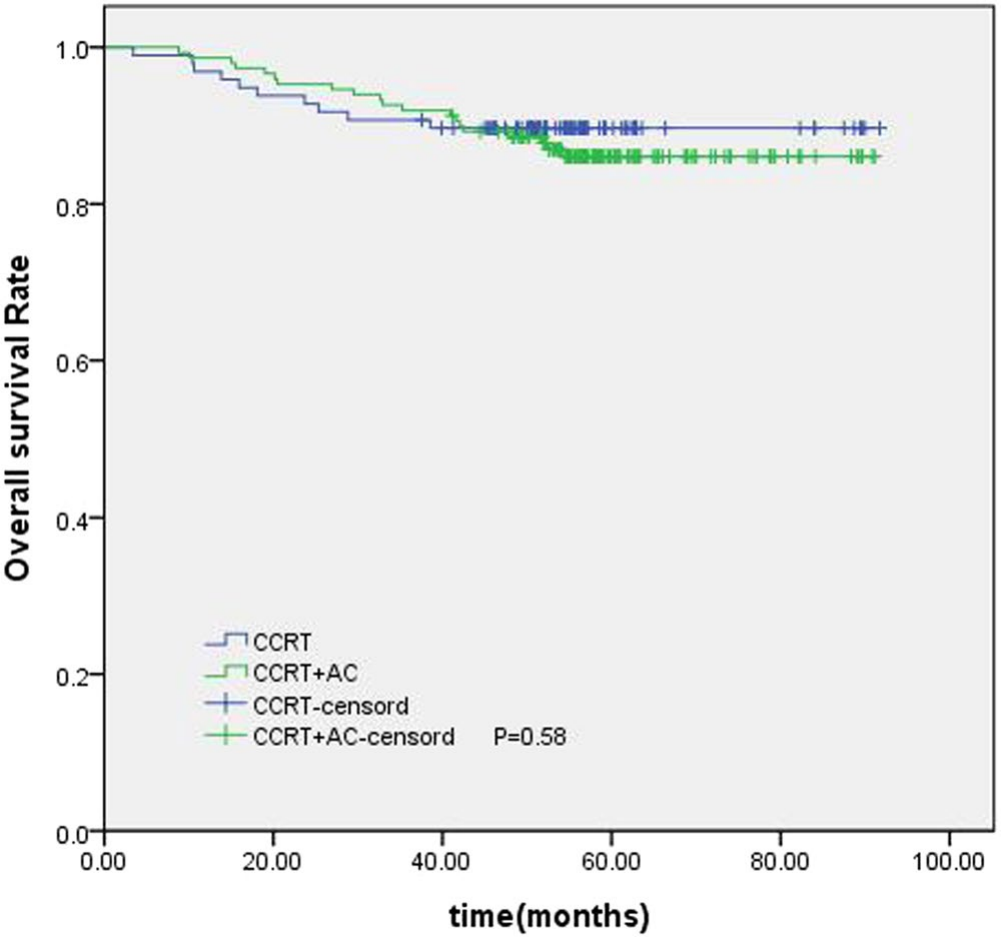
Kaplan-Meier survival curves for OS in patients of stage III with or without AC.

**Figure 10:**
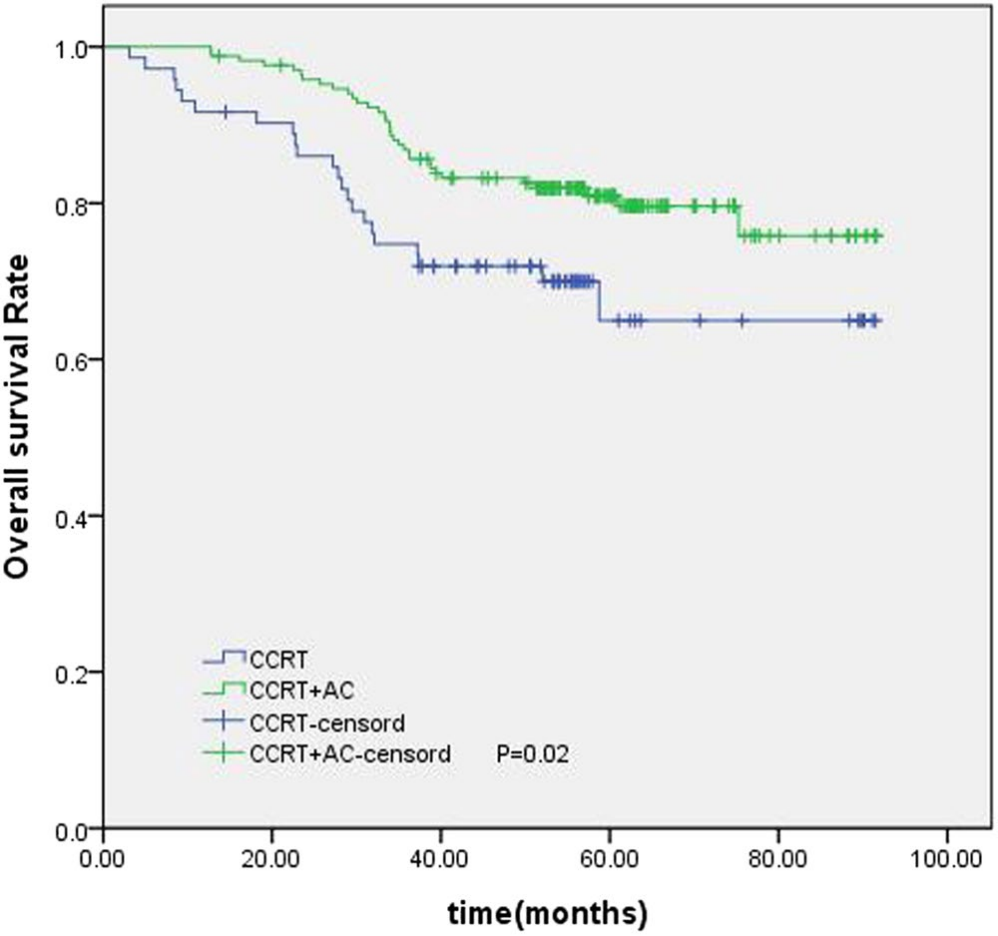
Kaplan-Meier survival curves for OS in patients of stage IV with or without AC.

The establishment of a nomogram prognostic model should not only assess the patient’s survival but, more importantly, provide guidance for the treatment. The clinical practice of locoregionally advanced NPC patients has involved the application of various chemoradiotherapy regimens. However, the optimal chemotherapy regimen and treatment plan are not standardized. The long-term results of a phase 3 multicenter randomized controlled trial showed that adjuvant chemotherapy failed to demonstrate a significant survival benefit for locoregionally advanced NPC patients^14^. However, some studies have conducted a stratified analysis and found that some patients with high-risk factors may benefit from adjuvant chemotherapy^15,16^. In our study, 461 patients underwent CCRT with or without AC; we were trying to find the patients who can benefit from adjuvant chemotherapy. According to the RPA-generated stratification based on OS, the patients were categorized into low, intermediate and high-risk groups. In subgroup analysis, the high-risk group benefited from adjuvant chemotherapy. Although adjuvant chemotherapy on its own did not significantly associate with survival for the entire cohort, it was significant for patients in the high-risk group (Fig. 6). Figures 7 and 8 show the Kaplan-Meier survival curves for low, intermediate, and high risk groups in patients without adjuvant chemotherapy and patients with adjuvant chemotherapy. Adjuvant chemotherapy improved OS in the high-risk group obviously. According to TNM staging, stage III patients do not benefit from adjuvant chemotherapy, stage IV patients can benefit from adjuvant chemotherapy (Figs. 9 and 10). Intergroup study 0099 reported that concurrent chemoradiotherapy followed by adjuvant chemotherapy delivered a significantly better 5-year OS benefit than radiotherapy alone (67% vs. 37%, respectively)^17^. The efficacy of CCRT-AC regimen was also studied by Lee *et al*.^18,19^ and Wee *et al*.^20^. However, controversy exists regarding whether NPC patients can benefit from adjuvant chemotherapy. A long-term results of a phase 3 multicentre randomised controlled trial reported that adjuvant cisplatin and fluorouracil chemotherapy still failed to demonstrate significant survival benefit after CCRT in locoregionally advanced NPC based on the long-term follow-up data, and addition of adjuvant cisplatin and fluorouracil did not significantly increase late toxicities^14^. Another two 10-year studies have confirmed that the addition of concurrent cisplatin plus adjuvant cisplatin-fluorouracil could significantly improve OS without excessive late toxicities for patients with regionally advanced NPC^19,21^. Li *et al*. undertook a network meta-analysis to establish the optimal chemotherapy strategy in advanced NPC, they found that CCRT + AC achieved better overall survival than CCRT (HR, 0.82; 95% CI, 0.67–1.00). CCRT + AC ranked best for overall survival^22^. In our opinion, not all locoregionally advanced NPC require adjuvant chemotherapy. A previous study of our team also confirmed that significant survival benefit of adjuvant chemotherapy after concurrent chemoradiotherapy in locally advanced high-risk nasopharyngeal carcinoma^23^. Liu *et al*. also reported adjvant chemtherapy can reduce distant failure and improve overall survival in high-risk NPC patients^15^. Currently, therapeutic decisions are based primarily on TNM stage. However, given tumor heterogeneity, similar stages patients have markedly different survival outcomes. The nomogram model established in this study can screen out high-risk groups requiring adjuvant chemotherapy and provide guidance for clinical treatment. These findings may help oncologists select therapeutic regimens for patients with loco-regionally advanced NPC. Further studies are warranted to determine the value of additional chemotherapy phases in specific patient subgroups.

Although this nomogram model demonstrated good levels of accuracy for the prediction of OS, there are some limitations that must be considered. First, our study was a retrospective design in a single center. Second, another potentially valuable prognostic factor (EBV DNA) was not be considered. Increasing research studies have confirmed that plasma EBV DNA is an important marker for survival^24–26^. However, this is not a routine examination item in most centers, and not all patients have these relevant data. As quantitative plasma EBV DNA assays conducted at different clinical laboratories yielded large variability in copy number without harmonization and the assay had not been standardized. In different laboratories, the number of copies of EBV DNA varies greatly, and there is no uniform standard at present. Standardization of EBV DNA serology assays is needed to allow for comparability of results obtained in different translational research studies across laboratories and populations^27^. Third, for linear variables that can be incorporated into multivariate regression models, such as age, it is necessary to further evaluate whether the nonlinear forms of these linear variables are more suitable for the inclusion model. When linear variables are transformed into nonlinear variables, percentile and other methods are commonly used to classify them. However, such classification is often subjective, important information may be lost, and selection bias may be introduced. To identify the least informative variables, we applied backward variable selection, with the intent of maximizing accuracy and promoting parsimony. This method yielded highly accurate and informative tools, which included only the key predictors without sacrificing accuracy or performance.

Additionally, the small sample size of patients analyzed could have possibly lowered the confidence levels of the validation derived from this study. Lastly, validation by a single institution does not provide strong evidence; a further large cohort, multi-institutional analysis is still required. We are conducting a lager cohort multi-centre clinical trial to establish a more specific prognostic model for nasopharyngeal carcinoma. external and prospective validation are needed.

Whether this nomograms can be applied to patients with distant metastasis or stage I-II, patients with two and three-dimensional conformal radiotherapy remains to be detemined.

## Conclusion

A nomogram composed of age, LDH, NLR, T-stage and N-stage provided statistically significantly better discrimination than the current TNM staging system. The clinical usefulness of the nomogram needs to be validated in prospective studies.

## Methods

The data of patients with newly diagnosed, nondisseminated, pathologically proven NPC who underwent IMRT and chemotherapy at the Affiliated Tumor Hospital of Guangxi Medical University between January 2010 and December 2012 were analyzed. Patients with a history of other malignancies and incomplete clinicopathologic data were excluded. All methods were in accordance with the relevant guidelines and regulations. This study was approved by the Ethics Committee of the Affiliated Tumor Hospital of Guangxi Medical University. All subjects have obtained informed consent.

### Collection of pretreatment baseline parameters

The following clinicopathological information was collected from each patient before treatment: sex, age, American Joint Committee on Cancer (AJCC) staging, LDH, SF, ALB, neutrophil count, lymphocyte count, radiotherapy dosimetry and type of chemotherapy. The NLR was calculated as the ratio of absolute counts between the peripheral neutrophil and lymphocyte measurements.

### Chemotherapy

Cisplatin (100 mg/m^2^) every three weeks was used for concurrent chemotherapy. Adjuvant chemotherapy included two or three cycles of cisplatin (80 mg/m^2^) on day 1 and 5-fluorouracil (750 mg/m^2^) daily for 4 consecutive days every four weeks.

### Radiotherapy

All patients completed IMRT as planned. The gross tumor volume (GTV) and cervical lymph node tumor volume (GTVnd) were defined as the gross extent of the tumor shown by CT/MRI and physical examinations. The clinical target volume (CTV1) included the GTVnx plus 5 to 10 mm margins (forward, both sides, up and down) and a 3 to 5 mm margin (back). The CTV2 included the GTVnd, the lymphatic regions, and the CTV1 with 5 to 10 mm margins (forward, both sides, up and down) and a 3 to 5 mm margin (back). The planning target volume (PTV) was defined as the CTV plus a margin of 3 mm depending on the proximity of critical structures. The radiotherapy prescription dose was PGTVnx 70~75.9 Gy/31~32 f, PGTVnd 60~73.6 Gy/30~32 f, PCTV1 60~68 Gy/30~31 f, and PCTV2 54~57.6 Gy/30~31 f.

### Follow up

All patients were assessed every 3 months during the first 2 years, every 6 months for the 3 subsequent years, and annually thereafter in clinic visits and telephone interviews. Physical examination, laboratory tests, and imaging were performed at every clinic visit. OS was measured from the date of diagnosis to the date of death or last follow-up, whichever occurred first.

### Statistical analysis

We included eight potential predictors in this analysis according to Harrell’s guidelines that the number of predictors should be less than ten times the number of deaths. Continuous variables were converted into categorical variables according to the median (age), and findings reported in previous studies (NLR^28^, LDH, SF and ALB^29^).

All statistical analyses were performed using SPSS 18.0 for Windows (SPSS, Chicago, IL) and R 3.4.3. Categorical variables were compared using the Chi-square test or Fisher’s exact test. Survival curves were depicted using the Kaplan-Meier method and compared using the log-rank test. Cox regression analysis was used for multivariate analyses. Proportional hazards assumptions were tested by using log minus log survival plots and time-by-covariate interactions^30^. A nomogram was formulated based on the results of multivariate analysis and by using the rms package in R version 2.14.1 (http://www.r-project.org/). A final model selection was performed by a backward step-down selection process using the Akaike information criterion (AIC)^31^. The performance of the nomogram was measured by the concordance index (C-index) and assessed by comparing nomogram-predicted versus observed Kaplan-Meier estimates of survival probability. Bootstraps with 1,000 resamples were used for these activities. Comparisons between the nomogram and the TNM staging system were performed with the rcorrp.cens package in Hmisc in R and were evaluated by the C-index. The larger the C-index, the more accurate the prognostic prediction was. During the external validation of the nomogram, the total points for each patient in the validation cohort were calculated according to the established nomogram. Then, Cox regression was performed on this cohort using the total points as a factor. Finally, the C-index and calibration curve were derived based on the regression analysis. P < 0.05 was considered statistically significant. RPA was applied to categorize the patients into 3 risk groups (low, intermediate, and high-risk) using the rpart package in R. The continuous NRI and IDIindex were used to determine whether the addition of independent predictors improved the predictionof survival.

## References

[1] Stiller CA (1994). International variations in the incidence of childhood carcinomas. Cancer epidemiology, biomarkers & prevention: a publication of the American Association for Cancer Research, cosponsored by the American Society of Preventive Oncology.

[2] Mao YP (2009). Re-evaluation of 6th edition of AJCC staging system for nasopharyngeal carcinoma and proposed improvement based on magnetic resonance imaging. International journal of radiation oncology, biology, physics.

[3] Lee AW, Ma BB, Ng WT, Chan AT (2015). Management of Nasopharyngeal Carcinoma: Current Practice and Future Perspective. Journal of clinical oncology: official journal of the American Society of Clinical Oncology.

[4] Xu C (2017). Establishing and applying nomograms based on the 8th edition of the UICC/AJCC staging system to select patients with nasopharyngeal carcinoma who benefit from induction chemotherapy plus concurrent chemoradiotherapy. Oral Oncol.

[5] Tang Lin-Quan, Li Chao-Feng, Li Jing, Chen Wen-Hui, Chen Qiu-Yan, Yuan Lian-Xiong, Lai Xiao-Ping, He Yun, Xu Yun-Xiu-Xiu, Hu Dong-Peng, Wen Shi-Hua, Peng Yu-Tuan, Zhang Lu, Guo Shan-Shan, Liu Li-Ting, Guo Ling, Wu Yi-Shan, Luo Dong-Hua, Huang Pei-Yu, Mo Hao-Yuan, Xiang Yan-Qun, Sun Rui, Chen Ming-Yuan, Hua Yi-Jun, Lv Xing, Wang Lin, Zhao Chong, Cao Ka-Jia, Qian Chao-Nan, Guo Xiang, Zeng Yi-Xin, Mai Hai-Qiang, Zeng Mu-Sheng (2015). Establishment and Validation of Prognostic Nomograms for Endemic Nasopharyngeal Carcinoma. Journal of the National Cancer Institute.

[6] Yang L (2015). Development and External Validation of Nomograms for Predicting Survival in Nasopharyngeal Carcinoma Patients after Definitive Radiotherapy. Sci Rep.

[7] Wu S (2015). Prognostic Nomogram for Patients with Nasopharyngeal Carcinoma after Intensity-Modulated Radiotherapy. PLoS One.

[8] Liang W (2016). Development and validation of a nomogram for predicting the survival of patients with non-metastatic nasopharyngeal carcinoma after curative treatment. Chin J Cancer.

[9] Zeng Q (2016). Nomograms for predicting long-term survival in patients with non-metastatic nasopharyngeal carcinoma in an endemic area. Oncotarget.

[10] Xia WX (2013). A prognostic model predicts the risk of distant metastasis and death for patients with nasopharyngeal carcinoma based on pre-treatment serum C-reactive protein and N-classification. European journal of cancer (Oxford, England: 1990).

[11] Untersteller K (2016). NT-proBNP and Echocardiographic Parameters for Prediction of Cardiovascular Outcomes in Patients with CKD Stages G2-G4. Clinical journal of the American Society of Nephrology: CJASN.

[12] Koller L (2017). Soluble Urokinase-Type Plasminogen Activator Receptor Improves Risk Prediction in Patients With Chronic Heart Failure. JACC. Heart failure.

[13] Lehmann Nils, Erbel Raimund, Mahabadi Amir A., Rauwolf Michael, Möhlenkamp Stefan, Moebus Susanne, Kälsch Hagen, Budde Thomas, Schmermund Axel, Stang Andreas, Führer-Sakel Dagmar, Weimar Christian, Roggenbuck Ulla, Dragano Nico, Jöckel Karl-Heinz, Meinertz T., Bode C., de Feyter P.J., Güntert B., Gutzwiller F., Heinen H., Hess O., Klein B., Löwel H., Reiser M., Schmidt G., Schwaiger M., Steinmüller C., Theorell T., Willich S.N., Bode C., Berger K., Figulla H.R., Hamm C., Hanrath P., Köpcke W., Ringelstein B., Dichgans M., Zeiher A. (2018). Value of Progression of Coronary Artery Calcification for Risk Prediction of Coronary and Cardiovascular Events. Circulation.

[14] Chen L (2017). Adjuvant chemotherapy in patients with locoregionally advanced nasopharyngeal carcinoma: Long-term results of a phase 3 multicentre randomised controlled trial. European journal of cancer (Oxford, England: 1990).

[15] Liu YC (2017). Prognostic impact of adjuvant chemotherapy in high-risk nasopharyngeal carcinoma patients. Oral Oncol.

[16] Xu T (2016). The role of adjuvant chemotherapy in nasopharyngeal carcinoma with bulky neck lymph nodes in the era of IMRT. Oncotarget.

[17] Al-Sarraf M (1998). Chemoradiotherapy versus radiotherapy in patients with advanced nasopharyngeal cancer: phase III randomized Intergroup study 0099. Journal of clinical oncology: official journal of the American Society of Clinical Oncology.

[18] Lee AW (2005). Preliminary results of a randomized study on therapeutic gain by concurrent chemotherapy for regionally-advanced nasopharyngeal carcinoma: NPC-9901 Trial by the Hong Kong Nasopharyngeal Cancer Study Group. Journal of clinical oncology: official journal of the American Society of Clinical Oncology.

[19] Lee AWM (2017). A multicenter, phase 3, randomized trial of concurrent chemoradiotherapy plus adjuvant chemotherapy versus radiotherapy alone in patients with regionally advanced nasopharyngeal carcinoma: 10-year outcomes for efficacy and toxicity. Cancer.

[20] Wee J (2005). Randomized trial of radiotherapy versus concurrent chemoradiotherapy followed by adjuvant chemotherapy in patients with American Joint Committee on Cancer/International Union against cancer stage III and IV nasopharyngeal cancer of the endemic variety. Journal of clinical oncology: official journal of the American Society of Clinical Oncology.

[21] Ng WT (2018). Concurrent-Adjuvant Chemoradiation Therapy for Stage III-IVB Nasopharyngeal Carcinoma-Exploration for Achieving Optimal 10-Year Therapeutic Ratio. International journal of radiation oncology, biology, physics.

[22] Li L (2019). Evolutionary role of chemotherapy in advanced nasopharyngeal carcinoma: a literature-based network meta-analysis. Cancer management and research.

[23] Liang ZG (2017). Significant survival benefit of adjuvant chemotherapy after concurrent chemoradiotherapy in locally advanced high-risk nasopharyngeal carcinoma. Sci Rep.

[24] Liu TB, Zheng ZH, Pan J, Pan LL, Chen LH (2017). Prognostic role of plasma Epstein-Barr virus DNA load for nasopharyngeal carcinoma: a meta-analysis. Clinical and investigative medicine. Medecine clinique et experimentale.

[25] Song Y (2017). The predictive value of pre- and post-induction chemotherapy plasma EBV DNA level and tumor volume for the radiosensitivity of locally advanced nasopharyngeal carcinoma. EXCLI journal.

[26] Liu LT (2015). The Prognostic Value of Plasma Epstein-Barr Viral DNA and Tumor Response to Neoadjuvant Chemotherapy in Advanced-Stage Nasopharyngeal Carcinoma. International journal of radiation oncology, biology, physics.

[27] Le QT (2013). An international collaboration to harmonize the quantitative plasma Epstein-Barr virus DNA assay for future biomarker-guided trials in nasopharyngeal carcinoma. Clinical cancer research: an official journal of the American Association for Cancer Research.

[28] Jiang Y, Qu S, Pan X, Huang S, Zhu X (2018). Prognostic value of neutrophil-to-lymphocyte ratio and platelet-to-lymphocyte ratio in intensity modulated radiation therapy for nasopharyngeal carcinoma. Oncotarget.

[29] Chen X (2017). Higher N stage and serum ferritin, but lower serum albumin levels are associated with distant metastasis and poor survival in patients with nasopharyngeal carcinoma following intensity-modulated radiotherapy. Oncotarget.

[30] Breslow N (1974). Covariance analysis of censored survival data. Biometrics.

[31] Harrell, F. E., Jr., Lee, K. L. & Mark, D. B. Multivariable prognostic models: issues in developing models, evaluating assumptions and adequacy, and measuring and reducing errors. *Statistics in medicine* **15**, 361–387, doi:10.1002/(sici)1097-0258(19960229)15:4<361::aid-sim168>3.0.co;2-4 (1996). 10.1002/(SICI)1097-0258(19960229)15:4<361::AID-SIM168>3.0.CO;2-4 8668867

